# The Comparative Effectiveness of Innovative Treatments for Cancer (CEIT-Cancer) project: Rationale and design of the database and the collection of evidence available at approval of novel drugs

**DOI:** 10.1186/s13063-018-2877-z

**Published:** 2018-09-19

**Authors:** Aviv Ladanie, Benjamin Speich, Florian Naudet, Arnav Agarwal, Tiago V. Pereira, Francesco Sclafani, Juan Martin-Liberal, Thomas Schmid, Hannah Ewald, John P. A. Ioannidis, Heiner C. Bucher, Benjamin Kasenda, Lars G. Hemkens

**Affiliations:** 1 Basel Institute for Clinical Epidemiology and Biostatistics, Department of Clinical Research, University Hospital and University of Basel, Spitalstrasse 12, 4031 Basel, Switzerland; 2 Swiss Tropical and Public Health Institute (Swiss TPH), Socinstrasse 57, Basel, 4002 Switzerland; 3 Univ Rennes, CHU Rennes, Inserm, CIC 1414 [(Centre d’Investigation Clinique de Rennes)], 22 rue Henri Le Guilloux, 35000 Rennes, France; 4 Department of Medicine, University of Toronto, 1 King’s College Circle, Toronto, M5S 1A8 ON Canada; 5 Department of Health Research Methods, Evidence and Impact, McMaster University, 1280 Main Street West, Hamilton, L8S 4K1 ON Canada; 6 Health Technology Assessment Unit, Institute of Education and Health Sciences, Oswaldo Cruz German Hospital, Rua João Julião, 245 1º andar, Bloco D, São Paulo, 01323-040 Brazil; 7 Department of Medicine, The Royal Marsden NHS Foundation Trust, Downs Road, Sutton, SM2 5PT Surrey UK; 8 Catalan Institute of Oncology (ICO) Hospitalet, Melanoma, Sarcoma and GU Tumors Unit, Av Gran Via de L’Hospitalet 199-203, Barcelona, 08908 Spain; 9 Vall d’Hebron Institute of Oncology (VHIO), Early Drug Development Unit (UITM), Pg Vall d’Hebron, 119-129, Barcelona, 08035 Spain; 10 St. Clara Hospital, Kleinriehenstrasse 30, Basel, 4058 Switzerland; 11 University Medical Library, University of Basel, Schönbeinstrasse 18-20, Basel, 4056 Switzerland; 12 Meta-Research Innovation Center at Stanford (METRICS), Stanford University, 1265 Welch Road, Stanford, 94305 CA USA; 13 Department of Medicine, Stanford University School of Medicine, 1265 Welch Road, Stanford, 94305 CA USA; 14 Department of Health Research and Policy, Stanford University School of Medicine, 1265 Welch Road, Stanford, 94305 CA USA; 15 Department of Biomedical Data Science, Stanford University School of Medicine, 1265 Welch Road, Stanford, 94305 CA USA; 16 Department of Statistics, Stanford University School of Humanities and Sciences, 1265 Welch Road, Stanford, 94305 CA USA; 17 Medical Oncology, University Hospital and University of Basel, Petersgraben 4, Basel, 4031 Switzerland

**Keywords:** US Food and Drug Administration (FDA), Drug regulation, Marketing authorization, Approval package, Drugs and biologics, Clinical trials, Cancer, Evidence synthesis, Systematic review

## Abstract

**Background:**

The available evidence on the benefits and harms of novel drugs and therapeutic biologics at the time of approval is reported in publicly available documents provided by the US Food and Drug Administration (FDA). We aimed to create a comprehensive database providing the relevant information required to systematically analyze and assess this early evidence in meta-epidemiological research.

**Methods:**

We designed a modular and flexible database of systematically collected data. We identified all novel cancer drugs and therapeutic biologics approved by the FDA between 2000 and 2016, recorded regulatory characteristics, acquired the corresponding FDA approval documents, identified all clinical trials reported therein, and extracted trial design characteristics and treatment effects. Herein, we describe the rationale and design of the data collection process, particularly the organization of the data capture, the identification and eligibility assessment of clinical trials, and the data extraction activities.

**Discussion:**

We established a comprehensive database on the comparative effects of drugs and therapeutic biologics approved by the FDA over a time period of 17 years for the treatment of cancer (solid tumors and hematological malignancies). The database provides information on the clinical trial evidence available at the time of approval of novel cancer treatments. The modular nature and structure of the database and the data collection processes allow updates, expansions, and adaption for a continuous meta-epidemiological analysis of novel drugs.

The database allows us to systematically evaluate benefits and harms of novel drugs and therapeutic biologics. It provides a useful basis for meta-epidemiological research on the comparative effects of innovative cancer treatments and continuous evaluations of regulatory developments.

**Electronic supplementary material:**

The online version of this article (10.1186/s13063-018-2877-z) contains supplementary material, which is available to authorized users.

## What is new

### Key findings

We established a comprehensive database on novel cancer drugs and therapeutic products approved by the US Food and Drug Administration (FDA) between 2000 and 2016. The current database will be used to describe the clinical trial evidence generated in the pre-marketing period, but the database can and will be updated and expanded for future meta-epidemiological analyses.

### What this adds to what is known

Publicly available drug approval documents offer highly valuable information that is very useful for evidence syntheses and research-on-research projects. The Comparative Effectiveness of Innovative Treatments for Cancer (CEIT-Cancer) database transparently describes and characterizes such information.

### What is the implication, what should change now

This database allows systematic analysis and assessment of early evidence on the benefits and harms of novel drug treatments and provides a solid basis for continuous meta-epidemiological analyses.

## Background

Cancer drug development is characterized by a perceived urgency to find novel treatments that improve patients’ survival and quality of life. Timely access to such beneficial treatments is considered paramount for patients with cancer. Before granting approval and market access, health authorities such as the FDA review the available evidence on benefits and harms from clinical trials and the claims made by the pharmaceutical companies and sponsors of the trials. The FDA examines the submitted clinical trial results, re-analyzes the trial’s patient-level data, and evaluates whether the trials were conducted and analyzed in accordance with the original study protocols [1, 2]. For drugs and therapeutic biologics that receive approval, the FDA reviews are made publicly available in the Drugs@FDA database as “approval packages” [3]. These packages provide a wealth of information on the evidence on benefits and harms of innovative treatments at the time of approval.

With the introduction of new incentives and approval pathways, the FDA aimed to facilitate the development and approval process of drugs intended to treat serious or life-threatening conditions, including cancer [4]. For example, some policies focus specifically on orphan drugs for rare diseases [4]. Between 2000 and 2012, 46 out of 47 oncology drugs approved by the FDA underwent expedited approval [5]. In 2012, a further policy for so-called “breakthrough” therapies was introduced for drugs with highly promising clinical evidence [5].

However, there is increasing discussion about the impact of these regulations because they may leave evidence gaps regarding efficacy and safety and increase uncertainty in clinical decision making as expedited and orphan drug approvals are often based on smaller studies than used in traditional approvals [6]. At the time of approval, there may be a dearth of evidence on hard clinical outcomes and subsequent follow-up evaluations suggest that such evidence either may never become available or may end up showing limited or no benefits [7–9]. Oncology and hematology are probably the medical fields which are currently most affected by such developments.

Numerous meta-epidemiological studies aimed to better understand the evidence at the time of approval of novel cancer drugs and therapeutic biologics using data from the FDA and the European Medicines Agency (EMA). We give an overview of these studies and the research in context in Table 1 (details of the underlying search strategy are provided in Additional file 1). The first related investigation that we are aware of was published in 2009 [10], and the number of publications peaked in 2017 with 10 articles. Nonetheless, a major limitation is that many of these studies cover only certain types of cancer (for example, solid tumors). Overall, there are four studies [10–13] which describe regulatory characteristics and clinical trials and assess endpoints and effect sizes used for approval on all cancer drugs, but none of them covers the most recently approved drugs (for example, after 2013). This would not allow the assessment of newer policies such as the breakthrough program introduced in 2012. Thus, the current knowledge on approval evidence for cancer drugs is marked by not only a limited scope but also a great diversity in methods and approaches, reducing the interpretability of the findings.

**Table 1:** Publications with similar or overlapping research questions

		Study characteristics				Characteristics described or analyzed			
(Reference, publication year)	Objective	Health authority	Time period	Approval type	Disease characteristics	Regulatory*	Trials	Endpoints	Effect sizes
Zeitoun et al. (2018) [28]	To characterize post-marketing trials of cancer drugs	EMA, FDA	2005–2010	Original and supplemental	Solid tumors and hematologic malignancies	x	x	x	
Barnes and Amir (2017) [29]	To describe the efficacy, safety, tolerability, and price of new cancer drugs.	FDA	2005–2016	Not described/Unclear	Solid tumors only		x	x	x
Booth and Del Paggio (2017) [30]	To evaluate the value of novel drugs using the ESMO Magnitude of Clinical Benefit Scale and ASCO Value Framework.	FDA	2015–2016	Not described/Unclear	Selected solid tumors ^[a]^		x	x	
Brooks et al. (2017) [31]	To understand the consequences of delaying approval of novel drugs until data on overall survival is available	FDA	1952–2016	Original and supplemental	Ten most common solid tumors ^[b]^			x	
Davis et al. (2017) [11]	To determine the availability of data on overall survival and quality of life benefits of cancer drugs.	EMA	2009–2013	Original and supplemental	Solid tumors and hematologic malignancies	x	x	x	x
Grossmann et al. (2017) [32]	To investigate the extent of EMA-approved cancer drugs that meet the threshold for “meaningful clinical benefit”, defined by the framework.	EMA	2011–2016	Original and supplemental	Solid tumors and hematologic malignancies		x	x	
Naci et al. (2017) [33]	To characterize preapproval and confirmatory clinical trials of drugs granted accelerated approval.	FDA	2009–2013	Original and supplemental	Solid tumors and hematologic malignancies ^[c]^		x	x	
Naci et al. (2017) [9]	To systematically evaluate the timing and characteristics of clinical trials of drugs receiving accelerated approval.	FDA	2000–2013	Original only	Any disease or medical condition	x	x	x	
Pease et al. (2017) [34]	To characterize controlled studies for drugs approved based on limited evidence.	FDA	2005–2012	Original only	Any disease or medical condition ^[d]^	x	x	x	
Salas-Vega et al. (2017) [35]	To evaluate the comparative therapeutic value of all new cancer medicines.	EMA, FDA	2003–2013	Original only	Solid tumors and hematologic malignancies			x	x
Smith et al. (2017) [36]	To characterize the primary endpoints used to support FDA approvals for new drug or novel hematologic malignancies indications.	FDA	2002–2015	Original and supplemental	hematologic malignancies only	x	x	x	x
Tibau et al. (2017) [37]	To derive the clinically meaningful benefit for FDA-approved drugs using the ESMO Magnitude of Clinical Benefit Scale.	FDA	2006–2016	Original and supplemental	Solid tumors only		x		
Grossmann and Wild (2016) [38]	To describe the knowledge about the clinical benefit of new cancer therapies at the time of approval.	EMA	2009–2016	Original and supplemental	Solid tumors and hematologic malignancies			x	x
Hoekman et al. (2015) [39]	To describe the marketing authorization of oncology medicines granted based on the conditional marketing authorization pathway	EMA	2006–2013	Original only	Solid tumors only	x	x	x	
Kim and Prasad (2015) [7]	To describe how often cancer drugs are approved based on a surrogate endpoint, whether subsequent studies for these drugs are reported, and whether the drugs improve overall survival.	FDA	2008–2012	Not described/Unclear	Solid tumors and hematologic malignancies	x		x	
Wang and Kesselheim (2015) [40]	To characterize the types of comparators and endpoints used in efficacy trials for approvals of supplemental indications, compared with the data supporting these drugs’ originally approved indications.	FDA	2005–2014	Supplemental only	Any disease or medical condition ^[e]^	x	x	x	
Winstone et al. (2015) [41]	To characterize the clinical trial evidence of orphan drugs.	EMA	2006–2014	Not described/Unclear	Solid tumors and hematologic malignancies^[f]^	x	x	x	
Downing et al. (2014) [6]	To characterize pivotal efficacy trials for newly approved novel therapeutic agents.	FDA	2005–2012	Original only	Any disease or medical condition ^[e]^	x	x	x	
Fojo et al. (2014) [42]	To determine the availability of data on overall survival and quality of life benefits of cancer drugs.	FDA	2002–2014	Not described/Unclear	Solid tumors only				x
Hartmann et al. (2013) [12]	To review the outcomes of marketing authorization applications for cancer drugs	EMA	2006–2011	Original only	Solid tumors and hematologic malignancies	x	x	x	x
Martell et al. (2013) [43]	To describe approval trends and characteristics.	FDA	1949–2011	Original and supplemental	Solid tumors and hematologic malignancies	x	x	x	
Thomas et al. (2013) [44]	To describe pre- and post-approval availability of published comparative efficacy studies.	FDA	2000–2010	Original only	Any disease or medical condition ^[g]^	x	x		
Goldberg et al. (2011) [45]	To quantify the availability of comparative efficacy data for new molecular entities.	FDA	2000–2010	Original only	Any disease or medical condition	x	x		
Johnson et al. (2011) [46]	To provide an overview of the regulatory history of accelerated approved oncology products.	FDA	1992–2010	Not described/Unclear	Solid tumors and hematologic malignancies^[c]^		x	x	
Kesselheim et al. (2011) [47]	To define characteristics of orphan cancer drugs and their pivotal clinical trials and to compare these with non-orphan drugs.	FDA	2004–2010	Not described/Unclear	Solid tumors and hematologic malignancies^[f]^	x	x	x	
Ocana and Tannock (2011) [48]	To determine if a difference in outcome between the experimental and control groups was detected that was equal to or greater than the value predefined in the protocol	FDA	2000–2010	Not described/Unclear	Solid tumors only			x	
Sridhara et al. (2010) [13]	To conduct an overview of products that were reviewed by the FDA’s Office of Hematology and Oncology Products for marketing approval and the regulatory actions taken during July 2005 to December 2007.	FDA	2005–2007	Original and supplemental	Solid tumors and hematologic malignancies	x	x	x	x
Tsimberidou et al. (2009) [10]	To review the long-term safety and efficacy or cancer drugs approved without evidence from randomized trials.	FDA	1973–2006	Original only	Solid tumors and hematologic malignancies^[h]^	x	x	x	x

This list is based on a systematic search (Additional file 1) but not intended to be exhaustive, as some relevant articles were brought to our attention by experts and could not be found with our limited search approach. *For example, approval pathways such as accelerated approval or orphan-drug status. Other regulatory characteristics (such as approval times, approval probabilities, or availability of pediatric label information) are not considered here. Abbreviations: *ASCO* American Society of Clinical Oncology, *EMA* European Medicines Agency, *ESMO* European Society for Medical Oncology, *FDA* US Food and Drug Administration. [a] Limited to breast, lung, colorectal, or pancreatic cancers. [b] Limited to breast, colorectal, endometrial, gastric, liver, pancreatic, prostate and renal cancer as well as melanoma and non-small-cell lung cancer. [c] Limited to drugs approved under accelerated approval. [d] Includes any drug approved on the basis of a single pivotal trial, pivotal trials that used surrogate markers of disease as primary endpoints or both. [e] Includes any drug. [f] Limited to orphan drugs only. [g] Limited to therapeutic biologics only. [h] Limited to drugs approved without evidence from randomized trials

To address such limitations, we intended to establish a comprehensive database allowing a continuous analysis of such regulatory developments in meta-epidemiological research. The ongoing “Comparative Effectiveness of Innovative Treatments for Cancer” (CEIT-Cancer) project aims to transparently describe and characterize the clinical trial evidence of novel cancer drugs. Our goal is to capture the relevant information required to systematically analyze and assess early evidence on benefits and harms of novel cancer drug treatments.

As a first step, we collected the pre-marketing clinical trial evidence using FDA approval documents with a specific focus on cancer drugs, randomized controlled trials (RCTs) and single-arm trials (SATs), and treatment effects on overall survival (OS), progression-free survival (PFS), and objective response rate (RR). However, the overall database structure is organized in a modular nature which allows continuous updating of the list of drugs, the addition of new variables, expansion of the number of topics, health authorities, and outcomes as well as linkage with other related datasets (for example, from post-approval evidence including non-randomized real-world studies).

Herein, we describe the rationale and design of the data collection process for the pre-approval evidence, including the organization of the data capture, the identification of clinical trial information, the assessment of trials for eligibility, and the data extraction.

## Methods

### Data collection

#### Project organization and database structure

The data collection consisted of three steps. In step 1, we made an inventory of novel FDA-approved drug products and acquired the corresponding FDA approval packages. In step 2, we made an inventory of RCTs and SATs reported in FDA approval documents, assessed their eligibility, and extracted trial design characteristics. In step 3, we extracted treatment effects on OS, PFS, and RR.

Steps 2 and 3 started with a planning and organizing phase (operationalization of concepts, drafting of an instruction manual for standardized data selection and extraction, setting up the extraction platform, pilot testing of the instruction manual and extraction platform, and training of reviewers) followed by an execution phase (independent data extraction and verification) and ended with a closing phase (documentation of activities). Specific project activities are described in greater detail in the following sections.

**Fig. 1:**
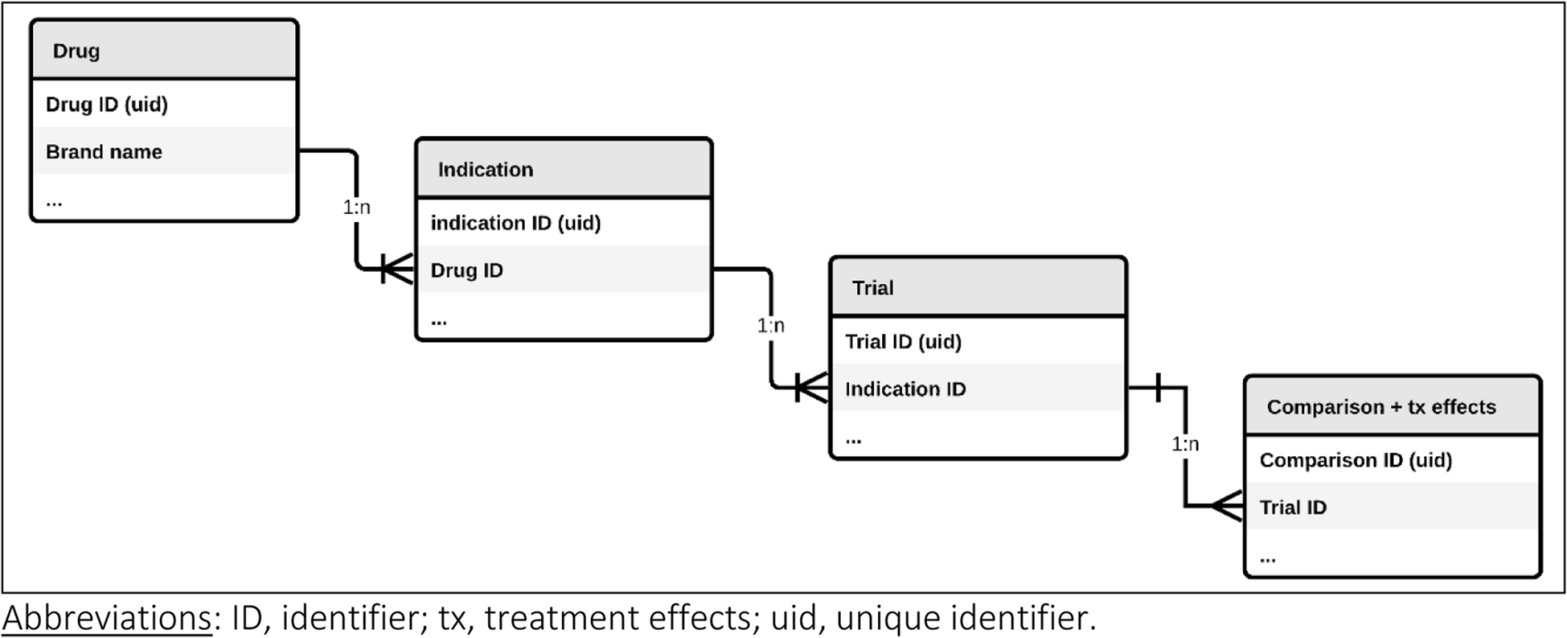
Database structure used in the Comparative Effectiveness of Innovative Treatments for Cancer (CEIT-Cancer) project for data collection and management

The clinical trial data were managed in a single database. The database consists of four data tables (with information about the drug, indication, trial, study groups and treatment comparisons, and treatment effects) that are linked in one-to-many (1:n) relationships (Fig. 1). The relational structure is indispensable because of the nature of the data (for example, multiple indications approved for a single drug, multiple clinical trials supporting approval of a single indication, and multiple comparisons within a single multi-arm clinical trial). We used both Microsoft Access as a local data extraction and management platform and Ragic [14] as a cloud-based equivalent.

#### Step 1: Inventory of FDA-approved drugs and acquisition of approval packages

The aims of this step were to identify and characterize all drugs licensed by the FDA for the treatment of cancer diseases and to download as well as prepare FDA approval documents for subsequent activities. This step was performed by a single reviewer (AL).

### Inventory of FDA-approved drugs

In a first stage, we created a list of novel drugs and therapeutic biologics (referred to in this article as “drugs”) that were granted their first FDA marketing authorization between 1 January 2000 and 31 December 2016. (Technically speaking, we included so-called “new molecular entities” and “new therapeutic biologics” approved via either a “New Drug Application” or a “Biologics License Application”.) The drug names were collected from the “Annual drug and biologic approval activity” reports for new molecular and biological entities (2000 to 2016) [15] as well as the “FDA reports on drug innovation” (2011 to 2016) [16]. Information on therapeutic biologics approved before 2004 is not available in these documents and therefore we reviewed the drug approval reports by month for the period of January 2000 to December 2003 obtained from the Drugs@FDA database [3].

### Selection of cancer indications

In a second stage, drugs were considered for inclusion in the CEIT-Cancer database if the original approval (that is, the first-ever approved use of a novel drug) was for the treatment of a solid tumor or hematological malignancy. Drugs without presumed cancer activity, such as supportive care drugs (for example, anti-emetics and hematopoietic stem cell mobilizing agents) or imaging drugs (for example, diagnostic radiopharmaceutical agents), were excluded. A medical oncologist (BK) was consulted in case of any doubts about eligibility.

### Extraction of information on drug, indication, and regulatory characteristics

In the third stage, we collated information on drug, indication, and regulatory characteristics for each eligible drug and cancer indication (“drug-indication pair”; Table 2). The line of treatment was determined by a medical oncologist (BK). The remaining information was retrieved from various information sources as follows.

**Table 2:** Variables collected in step 1 for each cancer drug-indication pair

Variable	(Data type), data value or code	Description and further elaboration
Drug characteristics		
Brand name	(Character string)	As accepted by the US Food and Drug Administration (FDA) and used in the US.
Generic name	(Character string)	According to US Adopted Names.
Type of active compound	“NME”; “NBE”	NME (New Molecular Entity, that is, a small molecule) or NBE (New Biologic Entity; that is, a biologic product).
Date of marketing authorization	(Date)	Format: YYYY-MM-DD.
Innovation class	“First-in-class”; “Advance-in-class”; “Addition-to-class”	Drug innovation class, following the definitions and categories described by Lanthier et al. [17]. New molecular or new biological entities are categorized as “First-in-class” if they define a new drug class, as “Advance-in-class” if they offer significant therapeutic advance (that is, they were granted priority review by the FDA) over existing drugs in the same class, or “Addition-to-class” in any other case.
Indication characteristics		
FDA-approved indication	(Character string)	Medical condition for which the drug of interest has been approved, according to the first-ever available FDA drug label.
Line of treatment	“1st”; “2nd”; “3rd”; “4th”	The clinical order the treatment is given
NDA/BLA number	(Integer)	FDA’s Original New Drug Application (NDA) or Biologics License Application (BLA) number. A unique identifier assigned to each application for approval submitted to the FDA.
Site of disease	“Breast”; “Digestive”; “Gastrointestinal”; “Endocrine and Neuroendocrine”; “Genitourinary”; “Gynecologic”; “Leukemia”; “Lymphoma”; “Musculoskeletal”; “Neurologic”; “Other - Multicentric Castleman’s Disease”; “Other - Other”; “T-cell malignancies”; “Respiratory/Thoracic”; “Skin”	Cancers by body location/system (following the classification by the National Cancer Institute (www.cancer.gov/types/by-body-location).
Regulatory characteristics		
Priority review	“Standard”; “Priority”	Priority review is an expedited FDA review program for drugs that provide a significant improvement over existing therapies.
Accelerated approval	“Yes”; “No”	Expedited FDA approval pathway for drugs that (a) treat serious conditions, (b) provide a meaningful advantage over available therapies, and (c) demonstrate effects on a surrogate endpoint that is reasonably likely to predict clinical endpoints. Accelerated approved drugs do not meet regulatory standards for traditional or full approval and are therefore required to provide evidence of clinical benefit in subsequent pivotal trials.
Breakthrough therapy designation	“Yes”; “No”	An expedited program at FDA introduced in 2012 for drugs that are (a) intended to treat serious conditions and (b) provide preliminary clinical evidence of substantial improvement over existing therapies.
Orphan designation	“Yes”; “No”	A status assigned by the FDA to rare disease indications if less than 200,000 people in the US are affected.

For drug-indication pair characteristics:

- “Annual drug and biologic approval activity” reports for new molecular and biological entities (2000 to 2016) [3], “FDA reports on drug innovation” (2011 to 2016) [16], and a peer-reviewed publication [17] for drug and regulatory characteristics, and
- the first-ever available FDA drug label from the Drugs@FDA database [3] for information about the FDA-approved indication(s).

For information on additional expedited programs and orphan status, we perused the following:

- “FDA reports on accelerated approvals” to identify accelerated approved indications [18]; that is, indications approved on the basis of preliminary evidence that does not meet regulatory standards for traditional (full) approval [4];
- “Breakthrough designation approval” reports [19] to identify indications that received a breakthrough therapy designation in the pre-approval period; that is, drugs that are expected to advance the treatment of certain diseases [4]; and
- FDA database of orphan drug product designations to identify indications that received an orphan status [20]; that is, drugs intended for the treatment of rare diseases affecting fewer than 200,000 people in the US [21].

All documents were downloaded or accessed on 2 November 2015 (for the 2000 to 2012 approvals) and 2 March 2017 (for the 2013 to 2016 approvals). We relied on the information from the Drugs@FDA database in the case of discrepant information between information sources (for example, if there were different approval dates presented). We categorized the drug innovation class (first-in-class, advance-in-class, and addition-to-class) in accordance with the algorithm of Lanthier et al. [17]. Accordingly, first-in-class drugs can be seen as “true” therapeutic innovation and define a new drug class. Advance-in-class drugs may offer an important therapeutic advance (that is, they were granted priority review by the FDA) over existing drugs in the same class. Drugs that do not fall under either of these two categories are categorized as addition-to-class.

### Approval packages

The FDA’s review of the pre-clinical and clinical information generated by a biopharmaceutical company during the course of drug development is summarized in FDA “approval packages” published in the Drugs@FDA database. We used a similar approach to retrieve the approval documents as described recently [22], and we provided practical details on how we navigated the documents elsewhere [23]. The following documents served as source documents throughout this project and were made suitable for text searching using Adobe Acrobat’s Optical Character Recognition (OCR) function:

- Medical review (sometimes referred to as clinical review)
- Statistical review
- Drug label
- Cross-discipline team leader review
- Summary review
- Multi-discipline review.

#### Step 2: Trial selection and characterization

The aims of this step were to identify eligible clinical trials in the medical review, assess their eligibility, and characterize their design characteristics. These activities were performed by teams of two independent reviewers. Trials include randomized and non-randomized studies (the latter within the category of SATs), and for each trial the database explicitly indicates whether a randomized design was used.

### Identification of trials, eligibility assessment, and data extraction

Each reviewer was provided with a set of indications to identify potentially eligible trials. Reviewers independently searched the medical review document for randomized trials as well as for trials that were indicated as pivotal for approval (that is, the trial was described as “approval”, “registration”, “major”, “pivotal”, or similar) regardless of whether they were randomized or not. For each trial, the reviewers recorded variables presented in Table 3. In particular, they extracted the study identifier, name, or acronym and determined whether the following criteria were met (each criterion was assessed separately):

1. the trial was explicitly described as pivotal to approval,
2. the patients were randomly assigned to treatment arms,
3. the patients matched broadly in their disease characteristics with the approved target population,
4. the patients were randomly assigned to at least one control arm that did not contain the drug under review (regardless of dose or administration schedule),
5. as per the judgment of the reviewer, a trial could still be relevant even if none of the abovementioned criteria was met; for example, if the trial is extensively discussed or the only trial evaluated in the medical review (which is sometimes the case in accelerated approval settings, where such trials are often not explicitly labeled as “pivotal” but extensively discussed in the documents).

**Table 3:** Variables collected in step 2 for trials that were randomized or explicitly labeled as pivotal

Variable	(Data type), data value or code	Description and further elaboration
Trial characteristics (for any trial identified in step 2)		
Trial name reference	(Character string)	Reference trial name.
Trial name 1	(Character string)	Alternative trial name 1.
Trial name 2	(Character string)	Alternative trial name 2.
Pivotal	“Yes”; “No”	Trial eligibility criteria: the trial is described as “pivotal” (or similar).
Randomized	“Yes”; “No”; “Single-arm”	Trial eligibility criteria: patients are randomly assigned to treatment arms.
On-label	“Yes”; “No”; “Partially”; “Not reported”	Trial eligibility criteria: the drug of interest is tested in the approved indication.
Comparator	“Yes”; “No”; “Partially”; “Not reported”	Trial eligibility criteria: the control intervention does not contain the active component of the drug under review.
Relevance	“Yes”; “No”	Trial eligibility criteria: two reviewers consider that this trial was definitely used for approval, but none of the abovementioned eligibility criteria are met.
Eligible rationale	“explicitly pivotal”; “likely pivotal”; “other pivotal”; “not eligible”	The rationale for trial eligibility based on eligibility algorithm.

After completion, the two independently generated datasets were compared and disagreements resolved by consensus. The inter-rater reliability for trial identification (as assessed with the Kappa statistic [24]) was good (74%). Ultimately, trials that met any of the following sets of criteria were deemed eligible:

- the trial was described as pivotal (criterion 1 alone is met; categorized as “explicitly pivotal”)
- the trial was not described as pivotal but was randomized (criterion 2), enrolled a population that matched the approved target population (criterion 3), and had a control arm that did not contain the intervention under review (criterion 4) (categorized as “likely pivotal RCT”)
- the trial was not “explicitly pivotal” or a “likely pivotal RCT” but considered otherwise essential (criterion 5) for the approval decision (categorized as “other pivotal”). Such trials were typically single-arm studies in accelerated approval settings.

For each eligible trial, teams of two independent reviewers extracted information on variables presented in Table 4.

**Table 4:** Variables collected in step 2 for eligible trials only

Variable	(Data type), data value or code	Description and further elaboration
Trial characteristics (for any trial deemed eligible in step 2)		
Randomization	“Yes”; “No”	Random allocation of patients to trial arms
N arms	(Integer)	The number of trial arms.
Other trial characteristics	“Parallel”; “Cross-over”; “Uncontrolled/historic control”	Patients are randomized to a concurrent control (“Parallel”) or to a sequence of treatments (“Cross-over”).
Comparison characteristics		
Arm 1		
Type	“Experimental”; “Active”; “Placebo”; “No treatment”; “Dose-comparison”	In add-on trials, comparators were categorized as “active” whenever an intervention given on top of an active treatment (for example, standard of care with or without placebo). Comparators were categorized as “No treatment” if “supportive therapy” or “usual care” was given which included a wide variety of treatments rather than a specific intervention.
Characteristics	(Character string)	All interventions in arm 1, including drug names, doses, and route of administration. Interventions used to avoid treatment-related complications were not recorded, such as pre-treatment with acetaminophen/diphenhydramine to reduce infusion reactions with intravenous infusion of therapeutic biologics, or anti-emetics to reduce nausea and vomiting associated with certain chemotherapies.
Arm 2		
Type	“Active”; “Placebo”; “No treatment”; “Dose-comparison”; “Uncontrolled/historic control”	See “Arm 1” above.
Characteristics	(Character string)	See “Arm 1” above.

#### Step 3: Treatment effect estimates on overall survival, progression-free survival, and response rate

The aim of this step was to retrieve treatment effect estimates on OS, PFS, and RR for each treatment comparison. This information was collected only for RCTs. This activity was performed by teams of two independent reviewers.

### Data extraction

We preferred trial analyses conducted by the FDA over sponsors’ analyses, whenever both were available. Similarly, more recent data cutoff dates were preferred over older cutoff dates if there were multiple analysis results on the same endpoint available. We used the statistical review document (or any other FDA approval documents) if the medical review document was not available or was incomplete or not legible.

For each treatment comparison, two reviewers independently searched the FDA review documents for treatment effect estimates on OS, PFS, and RR and extracted information on variables presented in Table 5. For OS and PFS endpoints with incomplete or missing information (for example, no confidence interval), we approximated treatment effect estimates following the methods described by Parmar et al. [25] and Tierney et al. [26]. At the end of the data collection activities in this step, the datasets of the two reviewers evaluating the same set of treatment comparisons were compared, and disagreements were resolved by consensus.

**Table 5:** Variables collected in step 3 for eligible randomized controlled trials retrieved on comparison level

Variable	(Data type), data value or code	Description and further elaboration
Overall survival OR progression-free survival		
Is the endpoint reported	“Yes”; “No”	.
Response criteria	(Character string)	Progression-free survival only: response criteria used to measure response to treatment
Number of patients in arm 1	(Integer)	Number of patients in arm 1 included in the endpoint analysis
Number of patients in arm 2	(Integer)	Number of patients in arm 2 included in the endpoint analysis
Number of events in arm 1	(Integer)	Number of patients with events in arm 1 included in the endpoint analysis
Number of events in arm 2	(Integer)	Number of patients with events in arm 2 included in the endpoint analysis
Hazard ratio: coverage probability	(Float)	Confidence level (1-alpha) in the endpoint analysis
Hazard ratio: point estimate	(Float)	Hazard ratio point estimate (selection rule: primary analysis according to the US Food and Drug Administration, but longest follow-up)
Hazard ratio: lower confidence bound	(Float)	The lower bound of the confidence interval of the hazard ratio estimate
Hazard ratio: upper confidence bound	(Float)	The upper bound of the confidence interval of the hazard ratio estimate
Randomization ratio	“1:1”; “Not 1:1”	Randomization ratio, extracted for incomplete endpoint effects to derive appropriate statistics [25, 26]
Regression *P* value	(Float)	Regression *P* value of the endpoint effect, extracted for incomplete endpoint effects to derive appropriate statistics [25, 26]
Test type	“1-sided”; “2-sided”; “Not reported”	One- or two-sided *P* value, extracted for incomplete endpoint effects to derive appropriate statistics [25, 26]
Hazard rate in arm 1	(Float)	Hazard rate in arm 1, extracted for incomplete endpoint effects to derive appropriate statistics [25, 26]
Hazard rate in arm 2	(Float)	Hazard rate in arm 2, extracted for incomplete endpoint effects to derive appropriate statistics [25, 26]
Logrank observed minus expected events in arm 1	(Integer)	Logrank Observed minus Expected (O-E) events in arm 1 (endpoint analysis), extracted for incomplete endpoint effects to derive appropriate statistics [25, 26]
Logrank observed minus expected events in arm 2	(Integer)	Logrank Observed minus Expected (O-E) events in arm 2 (endpoint analysis), extracted for incomplete endpoint effects to derive appropriate statistics [25, 26]
Logrank variance	(Float)	Logrank variance (endpoint analysis), extracted for incomplete endpoint effects to derive appropriate statistics [25, 26]
Median survival time in arm 1: point estimate	(Float)	Median survival time (point estimate) in arm 1
Median survival time in arm 1: lower confidence bound	(Float)	The lower bound of the confidence interval of the median survival time in arm 1
Median survival time in arm 1: upper confidence bound	(Float)	The upper bound of the confidence interval of the median survival time in arm 1
Median survival time in arm 2	(Float)	Median survival time (point estimate) in arm 2
Median survival time in arm 2: lower confidence bound	(Float)	Lower bound of the confidence interval of the median survival time in arm 2
Median survival time in arm 2: upper confidence bound	(Float)	Upper bound of the confidence interval of the median survival time in arm 2
Time unit	“Days”; “Weeks”; “Months”; “Years”; “Not reported”	Time unit used to measure median survival improvement
Tumor response		
Is the endpoint reported	“Yes”; “No”	.
Primary endpoint	“Yes”; “No”	Is the tumor response endpoint described as the primary endpoint of the trial
Type of hypothesis tested	“Superiority”; “Not 1° endpoint”; “Non-inferiority”	Is the trial designed to demonstrate the superiority of the test drug over control in tumor response
Response criteria	(Character)	Set of response criteria used to measure tumor response
Number of patients in arm 1	(Integer)	Number of patients in arm 1 included in the tumor response endpoint analysis
Number of patients in arm 2	(Integer)	Number of patients in arm 2 included in the tumor response endpoint analysis
Number of events in arm 1	(Integer)	Number of patients with events in arm 1 included in the tumor response endpoint analysis
Number of events in arm 2	(Integer)	Number of patients with events in arm 2 included in the tumor response endpoint analysis

## Discussion

We have successfully developed the CEIT-Cancer database, which transparently describes and characterizes information on the clinical trial evidence of novel cancer drugs at the time of their approval by the FDA.

Exploring characteristics of the evidence of novel cancer drugs at the time of their approval could greatly improve our understanding of the real-world clinical benefit and safety of such treatments. Importantly, it may also open new avenues of future research and regulation, leading to better-designed studies, reduced waste in research, and more rigorous criteria for health authorities and health systems to consider incorporating new interventions into the current cancer armamentarium.

The CEIT-Cancer database is a comprehensive, manually curated platform that captures regulatory, drug, indication, and clinical trial data from FDA approvals of novel cancer drugs. This database differs from previous investigations in three important ways. First, the CEIT-Cancer database covers a time frame of 17 years, substantially larger compared with most previous studies. Second, it assesses all types of cancers, including both solid tumors and hematologic malignancies. Third, the database encompasses the most recent FDA drug approvals. In addition, this database can be expanded to other medical fields and be linked with other databases. It can be augmented with post-approval evidence and also can be expanded for data extraction of approval documents from other health authorities, such as the EMA [11, 27].

We have set up the database and realized the project in a multidisciplinary team including experts in clinical trial methodology and conduct, clinical epidemiology, health technology assessment, biostatistics, clinical research, information management, public health, and medical oncology. The initial dataset covers a time period of 17 years. This allows us to investigate several regulatory developments over time and changes in the focus of drug development, such as the development of targeted agents and immunotherapy in contrast to classic cytotoxic chemotherapy. Following standardized and established data extraction procedures as in systematic reviews, we created a large evidence base on treatment effects and trial quality. This lays the foundation for our planned continuous meta-epidemiological analysis of novel drugs and therapeutic biologics within the CEIT-Cancer project. We are currently developing the infrastructure to make the database available and aim to obtain structural funding and support to provide a sustainable solution. Through the collaborating participation of other investigators, we aim to establish a data-sharing process to provide access to the database and foster further research.

## Conclusions

Publicly available drug approval documents offer highly valuable information that is very useful for evidence syntheses and research-on-research projects. The CEIT-Cancer database transparently describes and characterizes this information on the clinical trial evidence of novel cancer drugs. It allows systematic analysis and assessment of early evidence on benefits and harms of novel drug treatments in meta-epidemiological research. The modular nature and structure of the database as well as the data collection processes permit continuous updates and expansions. Overall, the database provides a solid basis for meta-epidemiological research of the evidence on novel treatments in cancer.

## Additional file

Additional file 1: Literature Search for Research in Context. (DOCX 13 kb)

## Abbreviations

CEIT-Cancer: Comparative Effectiveness of Innovative Treatments for Cancer
EMA: European Medicines Agency
FDA: US Food and Drug Administration
OS: Overall survival
PFS: Progression-free survival
RCT: Randomized controlled trial
RR: Response rate
SAT: Single-arm trial
