# Supplementary material for: The Comparative Effectiveness of Innovative Treatments for Cancer (CEIT-Cancer) project: Rationale and design of the database and the collection of evidence available at approval of novel drugs
Source: Trials. 2018 Sep 19;19:505. doi: 10.1186/s13063-018-2877-z (PMC6146631; doi:10.1186/s13063-018-2877-z)
Supplement: Supplementary file 1 — Literature Search for Research in Context. (DOCX 13 kb) [file 13063_2018_2877_MOESM1_ESM.docx]

# Additional file 1

Literature Search for Research in Context

| We searched PubMed with two strategies because we realized that the first search was not able to capture several pertinent articles. But even with the second strategy we have not identified some articles that were brought to our attention by experts. This is a pragmatic search approach as we did not hand-search articles, used additional databases or citation-search techniques because this would be beyond the scope of this supplementary information. We also used cancer-specific terms in the search strategy and focused on FDA and EMA. Overall, we aimed to capture most related articles and give an overview, but this can’t replace a complete systematic review with the intention to provide an exhaustive list of similar articles.  Titles and abstracts of references identified with the main search strategy were screened independently by two reviewers (LGH, BK). The titles of references identified with the second search were screened by one reviewer (AL). Full texts of all references deemed potentially eligible by any of the three reviewers in any search were assessed for eligibility by teams of two reviewers (AL, BK, or LGH).  We included articles describing a systematic analysis of approval evidence (general or cancer-specific) which reported specific analyses for oncological drugs and included a description of characteristics of (1) regulatory circumstances; (2) clinical trials; (3) endpoints; (4) or treatment effects. |
| --- |
| **First Search**  *Search date: 27 December 2017*  480 hits  Search terms:  (“Food and Drug”[tiab] OR United States Food and Drug Administration[mh] OR drugs@fda[tiab] OR “European medicines agency”[tiab] OR “Marketing Authorizations”[ti] OR “Marketing Authorisations”[ti] OR “Marketing Authorization”[ti] OR “Marketing Authorisation”[ti])  AND (oncology[tw] OR oncological[ti] OR "neoplasms"[MeSH Terms] OR neoplas*[ti] OR cancer*[ti] OR malignan*[ti] OR tumor[ti] OR tumour[ti] OR cancer[sb] OR anticancer[ti])  AND (APPROV*[tiab] OR PREAPPROVAL[tiab] OR Drug Approval[mh] OR PIVOT*[tiab])  AND (compar*[ti] OR Cross-Sectional[ti] OR Cross-Sectional Studies[mh] OR (Between January 19*[ti] OR Between February 19*[ti] OR Between March 19*[ti] OR Between April 19*[ti] OR Between May 19*[ti] OR Between June 19*[ti] OR Between July 19*[ti] OR Between August 19*[ti] OR Between September 19*[ti] OR Between October 19*[ti] OR Between November 19*[ti] OR Between December 19*[ti] OR Between January 20*[ti] OR Between February 20*[ti] OR Between March 20*[ti] OR Between April 20*[ti] OR Between May 20*[ti] OR Between June 20*[ti] OR Between July 20*[ti] OR Between August 20*[ti] OR Between September 20*[ti] OR Between October 20*[ti] OR Between November 20*[ti] OR Between December 20*[ti] OR between 19*[ti] OR between 20*[ti]) OR Meta-Analysis[ptyp] OR "Diffusion of Innovation"[mh] OR Cohort Studies[mh])  NOT ("Letter" [ptyp] OR ("Clinical Trial" [Publication Type] NOT "Clinical Trials as Topic"[mh]) OR ("animals"[MeSH Terms] NOT "humans"[MeSH Terms]))  OR drugs@fda[tiab] |
| **Second search**  *Search date: 07 January 2018*  11856 hits  Search terms:  ("Drug Approval"[Mesh] OR (approval[All Fields] OR approval'[All Fields] OR approval''[All Fields] OR approval's[All Fields] OR approvalall[All Fields] OR approvalethical[All Fields] OR approvalfor[All Fields] OR approvalformal[All Fields] OR approvalof[All Fields] OR approvalour[All Fields] OR approvalr[All Fields] OR approvals[All Fields] OR approvalsampling[All Fields] OR approvalsdata[All Fields] OR approvalsresearch[All Fields] OR approvalsthis[All Fields] OR approvalthe[All Fields] OR approvalthis[All Fields] OR approvalwe[All Fields] OR approvalwritten[All Fields]) OR ("licensure"[MeSH Terms] OR "licensure"[All Fields] OR "licensing"[All Fields]))  AND (("neoplasms"[MeSH Terms] OR "neoplasms"[All Fields] OR "cancer"[All Fields]) OR ("neoplasms"[MeSH Terms] OR "neoplasms"[All Fields] OR "oncology"[All Fields]) OR ("haematology"[All Fields] OR "hematology"[MeSH Terms] OR "hematology"[All Fields])) |
